# Coordinated elevation of membrane type 1-matrix metalloproteinase and matrix metalloproteinase-2 expression in rat uterus during postpartum involution

**DOI:** 10.1186/1477-7827-4-32

**Published:** 2006-06-02

**Authors:** Kengo Manase, Toshiaki Endo, Mitunobu Chida, Kunihiko Nagasawa, Hiroyuki Honnma, Kiyohiro Yamazaki, Yoshimitu Kitajima, Taeko Goto, Mika Kanaya, Takuhiro Hayashi, Toshihiro Mitaka, Tsuyoshi Saito

**Affiliations:** 1 Department of Obstetrics and Gynecology, Sapporo Medical University School of Medicine, South-1 West-16, Chuou-ku, Sapporo 060-8543, Japan; 2 Mica ladies Clinic, 5-21, Hiragishi-3jou-10, Toyohiraku, Sapporo 062-0933, Japan; 3 Department of Pathophysiology, Cancer Research Institute, Sapporo Medical University School of Medicine, Sapporo, Japan

## Abstract

**Background:**

The changes occurring in the rodent uterus after parturition can be used as a model of extensive tissue remodeling. As the uterus returns to its prepregnancy state, the involuting uterus undergoes a rapid reduction in size primarily due to the degradation of the extracellular matrix, particularly collagen. Membrane type-I matrix metalloproteinase (MT1-MMP) is one of the major proteinases that degrades collagen and is the most abundant MMP form in the uterus. Matrix metalloproteinase-2(MMP-2) can degrade type I collagen, although its main function is to degrade type IV collagen found in the basement membrane. To understand the expression patterns of matrix metalloproteinases (MMPs) in the rat uterus, we analyzed their activities in postpartum uterine involution.

**Methods:**

We performed gelatin zymography, northern blot analysis and immunohistochemistry to compare the expression levels of MT1-MMP, MMP-2, matrix metalloproteinase-9 (MMP-9) and the tissue inhibitors of MMPs-1 and 2 (TIMP-1 and TIMP-2) in the rat uterus 18 h, 36 h and 5 days after parturition with their expression levels during pregnancy (day 20).

**Results:**

We found that both MT1-MMP and MMP-2 localized mainly in the cytoplasm of uterine interstitial cells. The expression levels of MT1-MMP and MMP-2 mRNAs and the catalytic activities of the expressed proteins significantly increased 18 h and 36 h after parturition, but at postpartum day 5, their mRNA expression levels and catalytic activities decreased markedly. The expression levels of MMP-9 increased 18 h and 36 h after parturition as determined by gelatin zymography including the expression levels of TIMP-1 and TIMP-2.

**Conclusion:**

These expression patterns indicate that MT1-MMP, MMP-2, MMP-9, TIMP-1 and TIMP-2 may play key roles in uterine postpartum involution and subsequent functional regenerative processes.

## Background

During pregnancy, the uterus enlarges, which in rats is mainly caused by an increase in the amount of collagen and hypertrophy of the uterine smooth muscle cells. After parturition, the uterus undergoes involution during which it returns to its prepregnancy state. Matrix metalloproteinases (MMPs) are a group of structurally related endopeptidases that catalyze the degradation of various macromolecular components of the extracellular matrix and basement membrane [1,2], and induce various forms of tissue remodeling, including wound healing [3,4], trophoblast invasion [5,6], organ morphogenesis [7,8], and uterine [9-11], mammary gland [12,13], and prostate gland [14,15] involution. We previously reported that an increase in the expression levels of both membrane type 1-MMP (MT1-MMP) and MMP-2 plays a key role in tissue remodeling during corpus luteum structural involution both in rats and humans [16-18].

To obtain additional information on the activity of MMPs during uterine involution, we have initiated studies using a rat model to examine MMP expression and function in the uterus during pregnancy and after parturition. Although MT1-MMP is abundant in the uterus [19,20], little is known about its activity or that of MMP-2 during uterine involution. To the reason for this, we investigated the expression patterns of MT1-MMP, MMP-2, MMP-9, TIMP-1 and TIMP-2 and the activation of MMP-2 in the rat uterus during postpartum involution.

## Materials and methods

### Rat uterus

Pregnant Sprague-Dawley rats were obtained from Hokudo Co., (Sapporo, Japan) on day 17 of gestation, after which they were kept in our laboratory and maintained on a 12-hour light and 12-hour dark regimen (light 7:00–19:00) with free access to water and a standard diet. Uterine tissue for postpartum involution analysis was obtained from five rats per group on gestation day 20 and then 18 h, 36 h and 5 days after parturition. The Animal Care and Use Committee of the Sapporo Medical University School of Medicine approved all procedures of this study, which are in accordance with the standards described in the National Institutes of Health Guide for Care and Use of Laboratory Animals.

Each uterine tissue sample was divided into two pieces. One piece was fixed in 4% paraformaldehyde/PBS and embedded in paraffin for immunohistological analysis. The other was used for biochemical studies (zymography and northern blotting); all tissue samples were frozen on dry ice and then stored at -80°C until use.

### Chemicals

Ultraspec RNA was purchased from Biotex Laboratories, Inc. (Houston, TX); 3,3'-diaminobenzidine (DAB) was purchased from Katayama Chemical (Osaka, Japan); Nytran-Plus was purchased from Schleicher & Schuell (Keene, NH); 32P-dCTP and a Nick column were purchased from Amersham Pharmacia Biotech (Buckinghamshire, England); Prime-It II random primer labeling kits were purchased from Stratagen (La Jolla, CA); rabbit anti-rat MT1-MMP antiserum and anti-MMP-2 antibodies were purchased from Fuji Chemical Industries, Ltd. (Toyama, Japan); biotinylated antibodies and Vectastain ABC Elite kits were purchased from Vector Laboratories (Burlingame, CA); fetal calf serum (FCS) was purchased from Gibco (Grand Island, NY); APS-coated glass slides were purchased from Matsunami (Tokyo, Japan); STUF solution was purchased from Serotec Ltd. (Kidlington, Oxford, UK); and Block Ace was purchased from Dainippon Pharmaceutical Co. (Osaka, Japan).

### Northern blotting

Total RNA was extracted from uterine tissue samples using an Ultraspec RNA isolation system, after which the extracted RNA (20 μg/lane) was electrophoresed on 1% agarose/formaldehyde gels (100 V; 2 h), transferred overnight onto nylon membranes in 20 × SSC (3 M sodium chloride, 0.3 M trisodium citrate) and fixed using a UV linker. Filters were prehybridized for 4 h and then hybridized overnight at 42°C with a 32P-labeled cDNA probe. The probes used for Northern blotting were a 1.2-kb *Hind*-III- and *Eco*R-digested fragment of MT1-MMP cDNA, a 1.5-kb *Eco*RI- and *Bam*-HI- digested fragment of MMP-2 cDNA, a 0.6-kb *Cla*-I- and *Bam*-HI-digested fragment of TIMP-1 cDNA, and a 0.7-kb *Eco*-RI- and *Bg*-II-digested fragment of TIMP-2 cDNA [21]. A 450-bp cDNA fragment encoding the ribosomal protein L38 was used as an internal control [22].

The probes were radiolabeled with 32P-dCTP using Prime-It II random primer labeling kits, after which the labeled probes were purified on a Nick column before hybridization. After hybridization, the filters were washed four times: twice for 15 min each with 2 × SSC containing 0.1% SDS at room temperature, and twice for 15 min each with 0.2 × SSC containing 0.1% SDS at 65°C. The filters were then exposed to a Fuji RX X-ray film at -70°C for 1–2 days.

### Gelatin zymography

Gelatin zymography was carried out as previously described [16]. Briefly, uterine samples were homogenized (100 mg wet weight/ml of PBS containing 0.2% Triton X-100) using a Teflon glass tissue grinder on ice (15 strokes). The resulting homogenates were centrifuged at 12,000 × g for 20 min at 4°C, after which the supernatants were collected as the uterine extract. After assaying the protein concentration, aliquots of the extract (40 μg of protein) were electrophoresed in 10% polyacrylamide gels containing 1 mg/ml gelatin.

### Gelatin zymography using crude membrane fraction from rat uterus

The crude membrane fraction from the rat uterus was isolated as previously reported [17,23]. Briefly, uterine tissue samples were homogenized in 2 ml of Tris buffer containing 0.25 M sucrose using a Teflon glass tissue grinder at 45 × g on ice. The resulting homogenates were filtered through a nylon mesh (42 μm) and centrifuged at 120 × g for 10 min. The supernatant was then collected and centrifuged at 10,000 × g for 30 min at 4°C, and the pellet was collected as the crude membrane fraction and stored at -80°C until use. To examine the expression level of membrane-bound pro-MMP-2, aliquots of the crude uterine membrane fraction (20 μg) were mixed with 1 μl of FCS as a source of procollagenase and incubated for 2 h at 37°C. After terminating the procollagenase activity by adding of the sample buffer, the expression level of membrane-bound pro-MMP-2 as estimated from the liberated MMP-2 activity as determined by gelatin zymography.

### Immunohistochemistry

The uterine tissues embedded in paraffin were cut into 5-μm-thick sections and mounted on APS-coated glass slides. The sections were then deparaffinized with xylene and the slides were placed on a hot plate at 90°C, covered with a STUF solution for 10 min, and then rinsed several times with PBS. Endogenous peroxidase activity was blocked by incubating the slides in 0.6% H_2_O_2 _in methanol for 30 min at room temperature, after which Block Ace was applied for 30 min at room temperature to minimize nonspecific antibody binding. Primary antibodies were then applied to the sections for 60 min at room temperature, after which the sections were incubated with a biotinylated secondary antibody for 30 min. The sections were then stained using the ABC method with DAB as a substrate; hematoxylin and eosin stain was used as the counterstain. As a negative control, the slides were processed without incubation with primary antibodies. The sections were also stained with hematoxylin and eosin.

### Densitometry

Bands showing gelatinase activity were analyzed using NIH Image software (Version 1.61). Bands on Northern blots were analyzed using a BAS 2000 Bio-Imaging Analyzer (FUJI, Tokyo, Japan). Radioactivity was normalized to that in the L38 RNA band.

### Statistical analysis

Data are presented as mean ± SEM. The differences between groups were evaluated using one-way ANOVA with post hoc Schaffer's F-test and unpaired Student's t-test. Values of P < 0.05 were considered significant.

## Results

### Time-dependent localization of MMP-2 and MT1-MMP proteins in rat uterus

**Figure 1:**
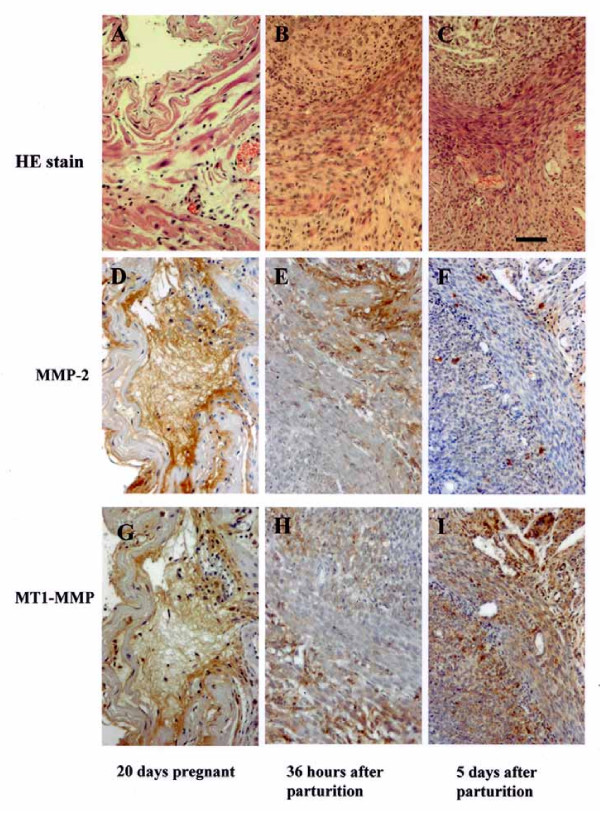
Immunohistochemical analysis of rat uterus. (Top row) Hematoxylin and eosin (HE) staining of rat uterus: (A) 20 days pregnant; (B) 36 h after parturition; (C) 5 days after parturition. (Middle and bottom rows) Immunohistochemical localizations of MMP-2 (middle) and MT1-MMP (bottom): (D, G) 20 days pregnant; (E, H) 36 h after parturition; (F, I) 5 days after parturition. Both MMP-2 and MT1-MMP are strongly stained in the interstitial cells at 36 h and weakly stained 5 days after parturition. Original magnification ×175, scale bar = 25 μm.

Immunohistochemical staining revealed that both MT1-MMP and MMP-2 proteins mainly localized in the cytoplasm of uterine interstitial cells 18 h (not shown) and 36 h after parturition, although uterine smooth muscle cells also showed weak MMP-2 protein staining (Fig. 1). Weak MMP-2 protein staining was also generally observed in the uterine tissue on day 5 after parturition, and a weaker MMP-2 protein staining was observed in the uterine tissue from 20-day pregnant rats.

### Expression levels of MMP-2, MT1-MMP, TIMP-1 and TIMP-2 mRNAs in rat uterus

**Figure 2:**
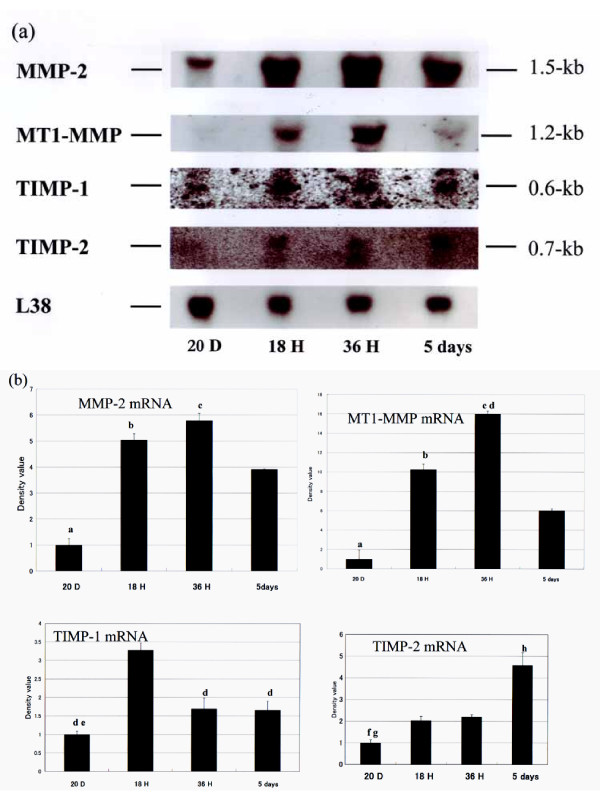
Expression levels of MT1-MMP, MMP-2, TIMP-1 and TIMP-2 mRNAs in rat uterus. (a) Representative Northern blot showing expression of indicated mRNA in uterine tissue harvested at indicated times. (b) Densitometric analysis of Northern blots showing relative mRNA expression levels of MMP-2, MT1-MMP, TIMP-1 and TIMP-2. Bars represent mean ± SEM (n = 5); a, P < 0.01 vs 18 h, 36 h and 5 days after parturition; b, P < 0.05 vs 5 days after parturition; c, P < 0.01 vs 5 days after parturition, d, P < 0.01 vs 18 h after parturition; e, P < 0.05 vs 36 h and 5 days after parturition; f, P < 0.05 vs 18 h and 36 h after parturition; g, P < 0.01 vs 5 days after parturition; h, P < 0.05 vs 18 h and 36 h after parturition. (20 days pregnant, 20D; 18 h after parturition, 18H; 36 h after parturition, 36H; and 5 days after parturition, 36 D)

As shown in Fig. 2, the uterine expression levels of both MMP-2 and MT1-MMP mRNAs time-dependently increased for at least 36 h after parturition, with larger increases observed in the MT1-MMP expression level. The expression levels then decreased markedly 5 days after parturition, although they remained above the levels expressed in the uterine tissue from pregnant rats. The expression level of TIMP-1 mRNA reached a peak within 18 h, the earliest time point measured. In contrast, the expression level of TIMP-2 mRNA increased only slightly 18 h and 36 h after parturition, but markedly higher expression levels were observed 5 days after parturition.

### Gelatinase activity in extracts of rat uterus

**Figure 3:**
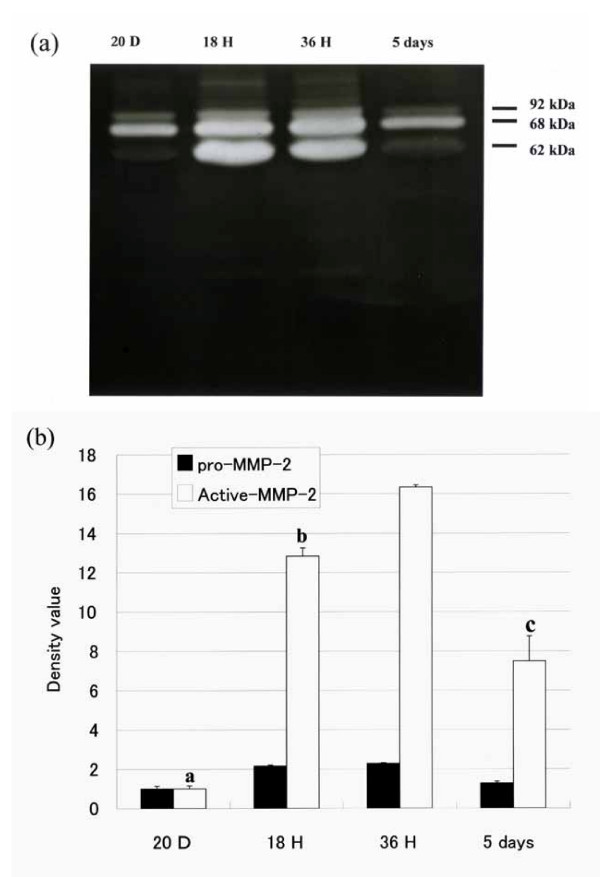
Zymography of homogenate extract from rat uterus. (a) Representative zymogram showing gelatinase activity of indicated enzyme in uterine tissue harvested at indicated times. Note that in the uterine tissues from pregnant rats, the expression of 68-kDa pro-MMP-2 predominated, whereas the expression of 62-kDa active MMP-2 predominated postpartum. (b) Densitometric analysis of zymograms showing relative gelatinase activity. Bars represent mean ± SEM (n = 5); a, P < 0.01 vs 18 h, 36 h and 5 days after parturition; b, P < 0.05 vs 36 h after parturition; c, P < 0.01 vs 18 h and 36 h after parturition (20 days pregnant, 20D; 18 h after parturition, 18H; 36 h after parturition, 36H; and 5 days after parturition, 36 D).

Gelatinase activity in uterine tissue extracts was examined by gelatin zymography (Fig. 3). In zymograms generated using uterine samples from pregnant (20 days) rats, most of the gelatinase activity was found in a 68-kDa band, with a lower activity in an approximately 62-kDa band. Treatment with EDTA/orthophenanthroline eliminated the activity, whereas pretreatment with amino phenyl mercuric acid, which is known to activate latent collagenase, elicited a marked shift in the activity from the 68-kDa band to the 62-kDa band (data not shown). Although not all MMPs exhibit gelatinase activity, which enables MMPs to be detected by zymography, we suspected that the major MMPs in the uterine tissue extracts from the pregnant rats were MMP-2 and MMP-9, which are present mainly as pro-MMP-2, the 68-kDa precursor of the 62-kDa activated form of MMP-2, and MMP-9, the 92-kDa. After parturition, however, the expression level of activated MMP-2 increased significantly over time corresponding to the expression level of MMP-2 mRNA, whereas the expression level of pro-MMP-2 remained unchanged (Fig. 3). The expression level of MMP-9 increased 18 h and 36 h after delivery.

### Expression levels of pro-MMP-2 in uterine plasma membrane fractions

**Figure 4:**
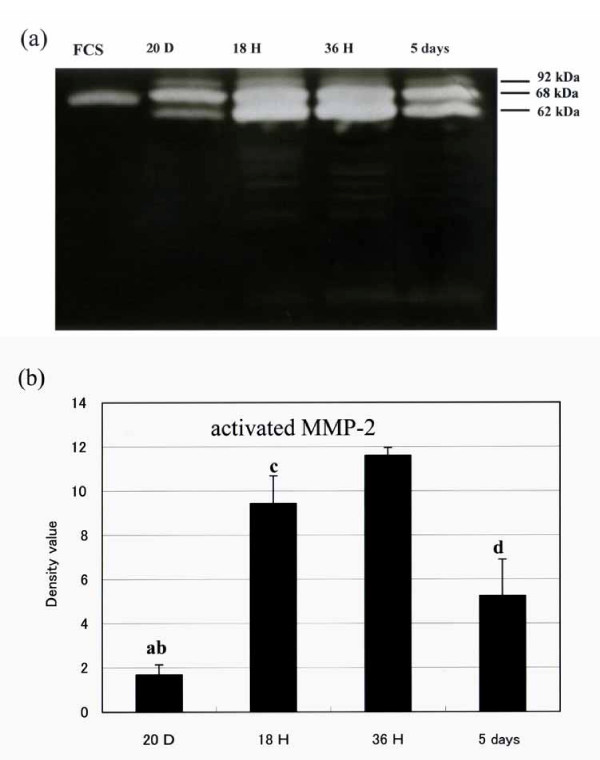
Expression level of membrane-bound Pro-MMP-2 in uterine tissue as revealed by zymography of crude uterine cell membrane fraction. (a) Representative zymogram obtained after exposing samples of crude uterine cell membrane fraction isolated from uterine tissue harvested at indicated times to FCS as source of procollagenase. (b) Densitometric analysis of zymograms showing relatively induced gelatinase activity. Bars represent mean ± SEM (n = 5); a, P < 0.01 vs 18 h and 36 h after parturition; b, P < 0.05 vs 5 days after parturition; c, P < 0.05 vs 36 h after parturition; d, P < 0.05 vs 18 h and 36 h after parturition (20 days pregnant, 20D; 18 h after parturition, 18H; 36 h after parturition, 36H; and 5 days after parturition, 36 D)

The expression levels of pro-MMP-2 in the plasma membrane fractions of the rat uterus were estimated from MMP-2 activity induced by incubation with FCS as a source of procollagenase and revealed by zymography. As shown in Fig. 4, the levels of expression and gelatinase activity of activated MMP-2 paralleled those of both MT1-MMP mRNA expression and the gelatinase activity of MMP-2 in the uterine tissue extract.

## Discussion

Immunohistochemical staining revealed that both MT1-MMP and MMP-2 proteins mainly localized in the cytoplasm of the uterine interstitial cells 18 h (not shown) and 36 h after parturition. Moreover, MMP-2 mRNA and MT1-MMP mRNA expressen levels time-dependently increased for at least 36 h. The level of the 62-kDa activated form of MMP-2 also increased 18 h and 36 h after parturition. The expression levels of gelatinase activity of the uterine tissue extract in the uterine plasma membrane fraction increased 18 h and 36 h after parturition. These results indicate that MMP-2 and MT1-MMP may play key roles in uterine postpartum involution

During pregnancy, the uterus is transformed into a large muscular organ sufficient to accommodate the fetus, placenta and amniotic fluid. After parturition, the uterus undergoes involution, a conspicuous feature characterized by the rapid decrease in the amount of collagen resulting from the extracellular degradation of collagen bundles. Activated collagenases cleave the collagen bundles into fragments, which then denature at body temperature into gelatin. Gelatinases then cleave the gelatin in small peptides that are rapidly removed in the blood stream [24]. To better understand the functions of MMPs in various uterine processes, we have initiated studies using a rat model for studying the activities of various MMPs in the uterus during pregnancy and after parturition. MT1-MMP, for example, has various functions that include the degradation of several types of collagen, the activations of other MMPs and activities related to apoptosis. This study showed that the expression levels of MT1-MMP and MMP-2 were significantly increased in the cytoplasm of uterine interstitial cells in the first five days after parturition, which suggests that these two enzymes play important roles in the postpartum involution of the enlarged uterus in rats. Various of MMPs are reportedly involved in tissue remodeling during postpartum uterine involution. Stygar et al. reported that cervical stromal fibroblasts and smooth muscle cells were identified as the main sources of MMP-2, whereas MMP-9 protein was observed only in invading leukocytes. MMP-2 and MMP-9 are involved also in the cervical ripening process [25]. Lyons et al. reported that MMP-9 may be involved in preterm labor, cervical maturity, and postpartum uteruine involution [26]. Roh et al. reported that the proMMP-9 in the uterine tissue maybe related to the remodelling of human myometrium during labor [27]. We also consider that MMP-9 is related to postpartum uterine involution from the results of our gelatine zymograpy experiment (Fig. 3(a)).

Evidence suggests that MT1-MMP causes the degradation of collagen types I, IV and V and elastin [28,29], and activates membrane-bound pro-MMP-2 [30-33]. Although it is thought that the main function of the activated MMP-2 is the degradation of type IV collagen in the basement membrane, it also cleaves type I collagen [34], which is consistent with the finding that during postpartum uterine involution, MT1-MMP and MMP-2 act together to degrade the abundant type I collagen.

We found that the time courses of the expressions of MMP-2, TIMP-1 and TIMP-2 differed from those described in the reports mentioned above. This difference is reflected in part by the transiently high expression level of TIMP-1 observed 18 h after parturition. Because TIMP-1 is known to bind stoichiometrically to almost all MMP active sites, thereby irreversibly inhibiting the enzyme's activity [33,35], its upregulation would be expected to mitigate the effect of the abrupt concurrent increase in MMP expression level. On the other hand, the suppression of TIMP-1 transcription 36 h after parturition would likely contribute to the maintenance of a high MMP activity.

In contrast to TIMP-1, TIMP-2 transcription was enhanced only slightly 5 days after parturition. Although TIMP-2 exerts a strong inhibitory effect on MMPs similar to TIMP-1 [36], it also forms MT1-MMP/TIMP-2 and MT1-MMP/TIMP-2/pro-MMP-2 complexes, which are involved in the activation of pro-MMP-2 [37-39]. This suggests that at 18 h and 36 h postpartum the slight increase in TIMP-2 expression level induced proMMP-2 activation. In contrast, the much larger increase in TIMP-2 expression level observed 5 days after parturition, which coincides with the decrease in the activities of MT1-MMP and MMP-2, mediated pro-MMP-2 suppression. Taken together, these findings strongly suggest that MT1-MMP, MMP-2 and MMP-9 are time-dependently regulated and play important roles in tissue remodeling during postpartum uterine involution.
